# Targeted Inactivation of GPR26 Leads to Hyperphagia and Adiposity by Activating AMPK in the Hypothalamus

**DOI:** 10.1371/journal.pone.0040764

**Published:** 2012-07-16

**Authors:** Daohong Chen, Xiaolei Liu, Weiping Zhang, Yuguang Shi

**Affiliations:** 1 Department of Cellular and Molecular Physiology, Pennsylvania State University College of Medicine, Hershey, Pennsylvania, United States of America; 2 Department of Pharmacology, School of Pharmaceutical Sciences Central South University, Changsha, China; 3 Department of Pathophysiology, Second Military Medical University Shanghai, China; Pennington Biomedical Research Center, United States of America

## Abstract

G-protein coupled receptor 26 (GPR26) is a brain-specific orphan GPCR with high expression in the brain region that controls satiety. Depletion of GPR26 has been shown to increase fat storage in *C. elegans*, whereas GPR26 deficiency in the hypothalamus is associated with high genetic susceptibility to the onset of obesity in mice. However, the metabolic function of GPR26 in mammals remains elusive. Herein, we investigated a role of GPR26 in regulating energy homeostasis by generating mice with targeted deletion of the *GPR26* gene. We show that GPR26 deficiency causes hyperphagia and hypometabolism, leading to early onset of diet-induced obesity. Accordingly, GPR26 deficiency also caused metabolic complications commonly associated with obesity, including glucose intolerance, hyperinsulinemia, and dyslipidemia. Moreover, consistent with hyperphagia in GPR26 null mice, GPR26 deficiency significantly increased hypothalamic activity of AMPK, a key signaling event that stimulates appetite. In further support of a regulatory role of GPR26 in satiety, GPR26 knockout mice also demonstrate hypersensitivity to treatment of rimonabant, an endocannabinoid receptor-1 antagonist commonly used to treat obesity by suppressing appetite in humans. Together, these findings identified a key role of GPR26 as a central regulator of energy homeostasis though modulation of hypothalamic AMPK activation.

## Introduction

Obesity and its associated metabolic diseases represent the most common health risks in developed nations, and have emerged as a major health issue in many developed countries due to sedative life style and consumption of Western diet enriched with animal fat [1]. In the United States, obesity has become an epidemic with alarming rate of increase. Currently, more than 30% of the US populations are obesity and more than 40% of adults are considered overweight or obese. Another major concern is the rising rate of obesity in children and adolescents, with more than 16% of them are obese, and its prevalence rates are steadily growing in young people. Besides, there has been a biomedical consensus that obesity significantly increases the risk of a number of chronic disorders including type 2 diabetes, coronary artery disease, hypertension, fatty liver disease, and several types of cancers [2]–[5].

Although the etiology of obesity is poorly understood, it has been realized that central in the pathogenesis of obesity is a chronic positive energy balance resulted from increased caloric intake or/and decreased energy expenditure. The neuro-endocrine system plays a pivotal role in regulation of energy homeostasis, in which G protein-coupled receptor (GPCR) pathways are increasingly discovered to be an important modulator [6]–[8]. GPR26 is a central orphan GPCR whose biological function remains elusive. GPR26 consists of a protein with 317 amino acids and is most closely related to the serotonin receptor 5-HT_5A_ and gastrin releasing hormone BB2 receptor, suggesting a possible role in regulating energy homeostasis. In support of this hypothesis, GPR26 is most abundantly expressed in ventromedial hypothalamic nucleus and cortex [9], [10]. Furthermore, depletion of an GPR26 homolog mediated by genome-wide RNA interference (RNAi) in *C. elegans* resulted in increased body fat storage [11]. However, the physiological importance of GPR26 in metabolism, if any, remains unknown in mammals.

In the study, we investigate a possible role of GPR26 in energy homeostasis by generating mice with targeted deletion of the *GPR26* gene. We show that mice with GPR26 deficiency exhibit hyperphagia and decreased energy expenditure, leading to high propensity to diet-induced obesity and its related metabolic complications. Consistent with the findings, GPR26 deficiency significantly increased phosphorylation of AMPK at ser172, a major activation site that is implicated in hyperphgia and onset of obesity. Our findings identified for the first time a key role of GPR26 in energy homeostasis, suggesting that targeting GPR26 with chemical compounds may provide a novel treatment for obesity thought modulation of appetite.

## Results

### Generation of Mice with Targeted Deletion of the GPR26 Gene

**Figure 1:**
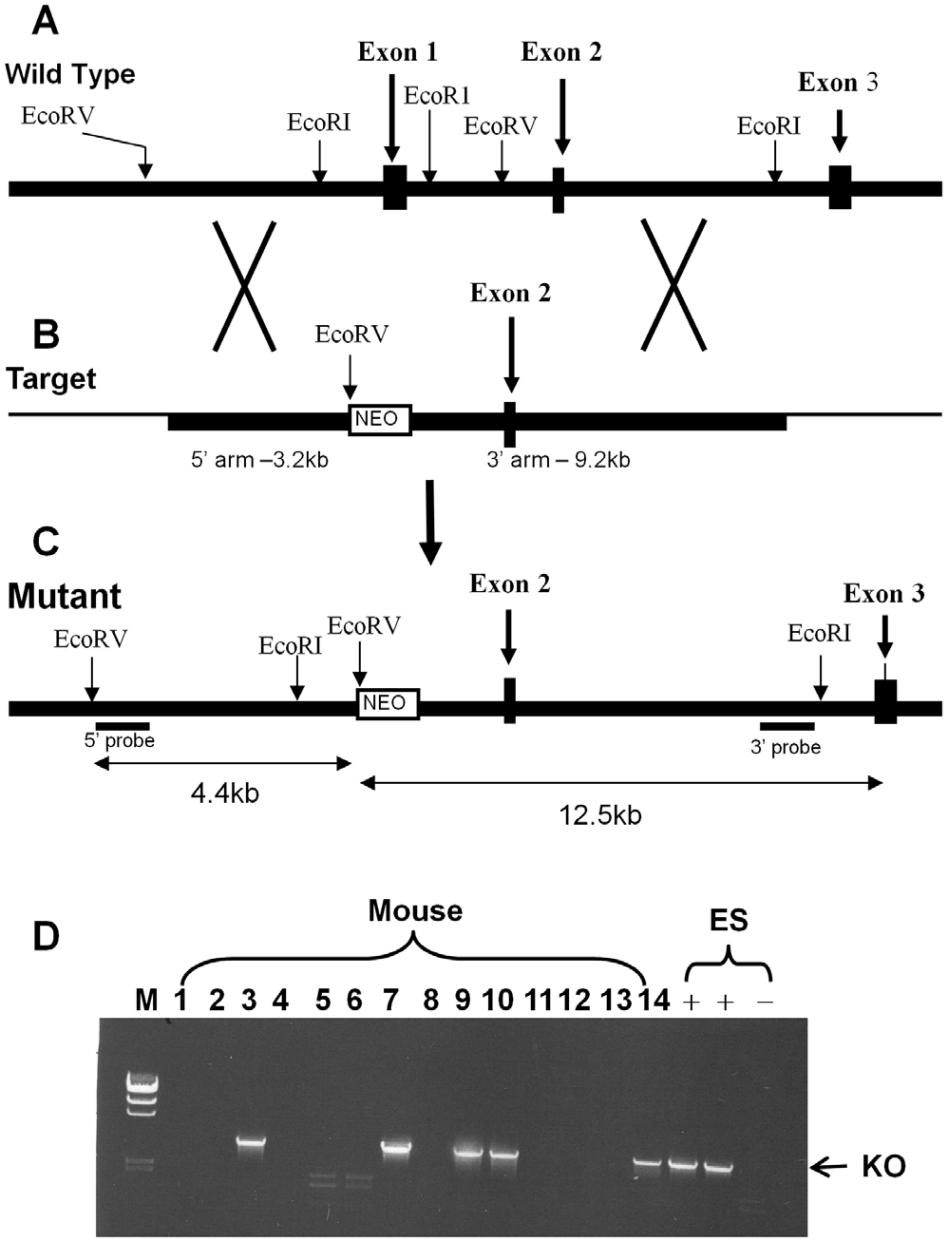
Generation of mice with targeted inactivation of the GPR26 gene. (**A**), a diagram depicting the genomic structure of the mouse *GPR26* gene. (**B**), the targeting vector used to delete the first exon of the *GPR26* gene. (**C**), the structure of the expected mutant allele with deletion of the first exon of the *GPR26* gene. (**D**), a representative PCR screening result of positive ES clones and offspring with targeted deletion of GPR26, as identified by the presence of a ∼3.8 kb band (indicated with the arrow).

To determine the physiological functions of GPR26 *in vivo*, we generated mice deficient in GPR26 expression by homologous recombination in ES cells. We engineered a targeting vector to replace the first exon of the coding sequence of GPR26 with a neomycin resistance cassette (Fig. 1). A targeted mutant ES clone identified by PCR and confirmed by Southern blot was injected into C57BL/6 blastocysts and resulted in chimeric littermates that transmitted the disrupted GPR26 allele through the germline transmission. Heterozygous mutant mice were intercrossed to generate homozygous GPR26 mutants that were identified by PCR and confirmed by Southern blot analyses of genomic DNA.

### GPR26^−/−^ Mice Demonstrate an Increased Adiposity and Hyperglycemia

**Figure 2:**
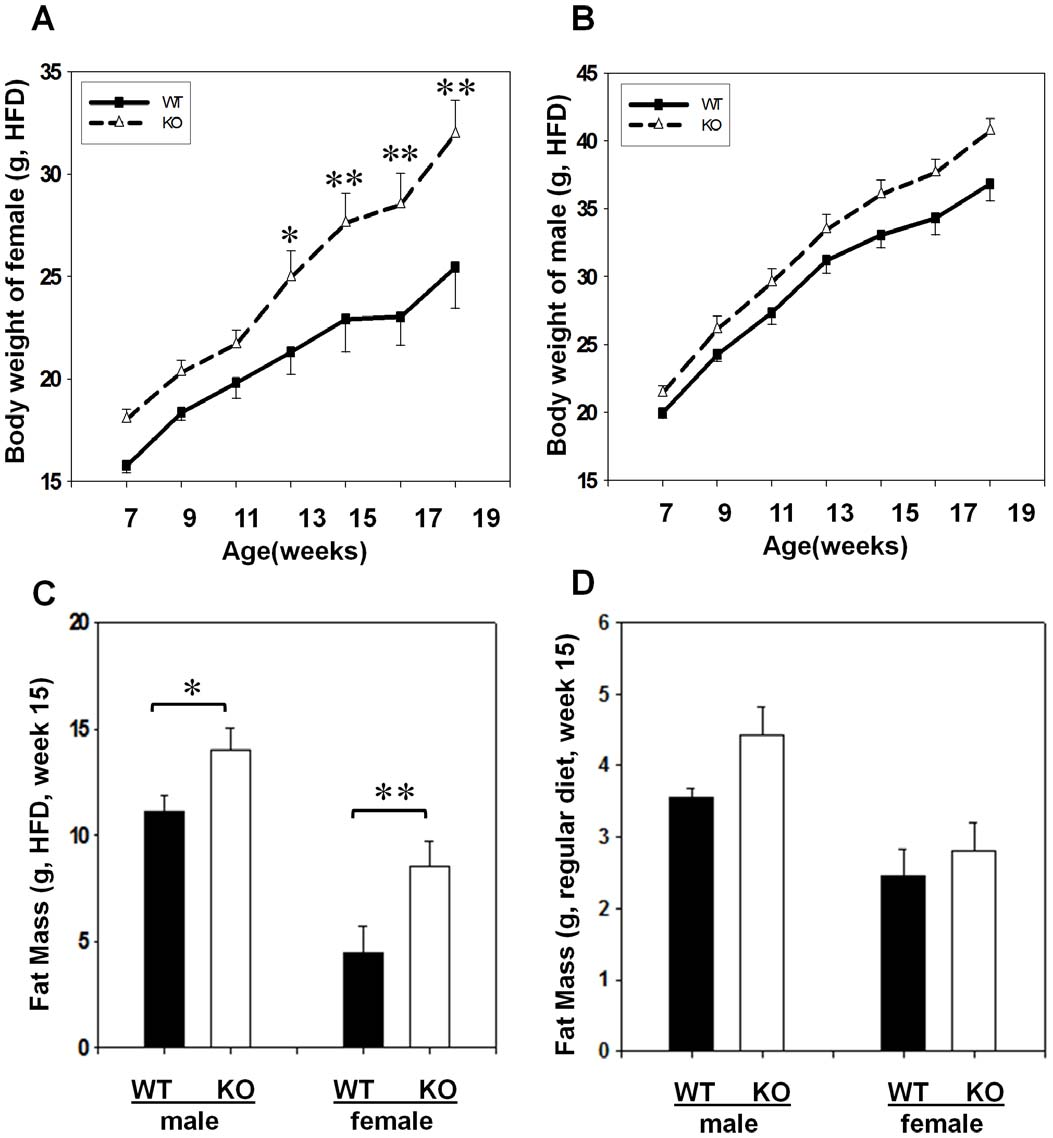
GPR26 deficiency causes early onset of diet-induced obesity. GPR26^−/−^ mice (KO) and the wild type controls (WT) at the 7 weeks of age were fed a regular chow or a high fat-diet (HFD) for 12 consecutive weeks. (**A–B**), body weight were analyzed weekly from female (**A**) and male (**B**) mice during 12 weeks of high fat feeding. (**C–D**), fat masses were analyzed after 12 weeks of high fat feeding (**C**) or regular chow (**D**) by quantitative nuclear magnetic resonance (QMR) using an Echo System instrument. N = 8, *p<0.05, **p<0.01 when compared with wild type controls.

The GPR26^−/−^ mice were born at the predicted Mendelian ratios without any obvious phenotypic abnormality at three months of age when fed a standard mouse chow (data not shown). However, after feeding on the high-fat diet which contains 40% calories from animal fat for 12 consecutive weeks, the weight gain in GPR26^−/−^ mice (KO) was significantly higher in female (Fig. 2A), but not in male mice (Fig. 2B), than the wild type control littermates (WT). The difference was caused by increased fat mass in GPR26^−/−^ mice as measured by ^1^H-nuclear magnetic resonance (Fig. 2C). The total body fat content was significantly higher in female GPR26^−/−^ than wild type controls. In contrast, such a difference was diminished when fed a regular diet (Fig. 2D), which is consistent with a lack in body weight differences between GPR26 and the wild type controls when fed a regular chow.

**Figure 3:**
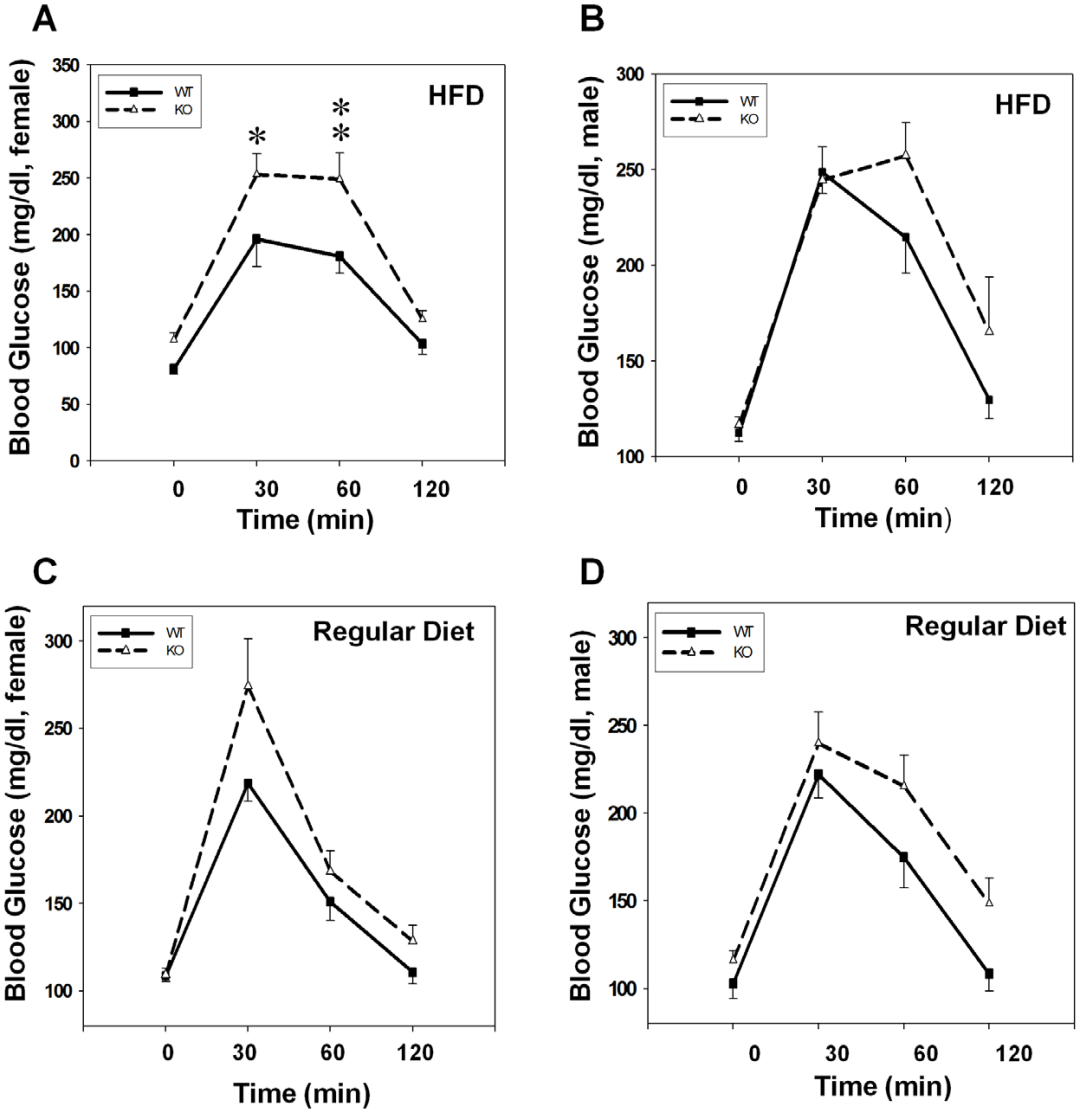
GPR26-deficicent mice exhibit glucose intolerance. Oral glucose tolerance tests were carried out in GPR26^−/−^ mice (KO) and the wild type controls (WT) after 12 weeks of high-fat diet (HFD) or regular chow. Mice were orally gavaged with 2.0g of glucose per kg of body weight. Mouse tail blood samples were collected at indicated time points and analyzed for blood glucose levels by the ACCU-CHEK Blood Glucose Meter. (**A–B**): blood glucose levels of female (**A**) and male (**B**) mice on high-fat diet; (**B–D**): blood glucose levels of female (**C**) and male (**D**) mice on regular diet. N = 8, *p<0.05, **p<0.01 when compared with wild type controls.

Obesity significantly increased the risk of the development of type 2 diabetes by impairing glycemic controls. We next determined whether increased adiposity in GPR26 deficient mice would impair glucose homeostasis by oral glucose tolerance test. The results show that after an overnight fast, blood glucose levels were slightly higher in female GPR26^−/−^ mice than the wild type controls (Fig. 3A). However, blood glucose levels in female GPR26^−/−^ mice were significantly higher than those in the wild type control mice during oral glucose tolerance test, suggesting glucose intolerance (Fig. 3A). Consistent with a lack of changes in body weight in male GPR26^−/−^ mice, the blood glucose levels were indistinguishable between male GPR26 deficient mice and their male wild type controls (Fig. 3B). Likewise, there were no significant differences in blood glucose level or glucose tolerance levels between the GPR26 null mice and the wild type controls in both genders when fed a regular chow diet (Fig. 3C&D).

### Hyperphagia and Decreased Energy Expenditure in GPR26^−/−^ Mice

**Figure 4:**
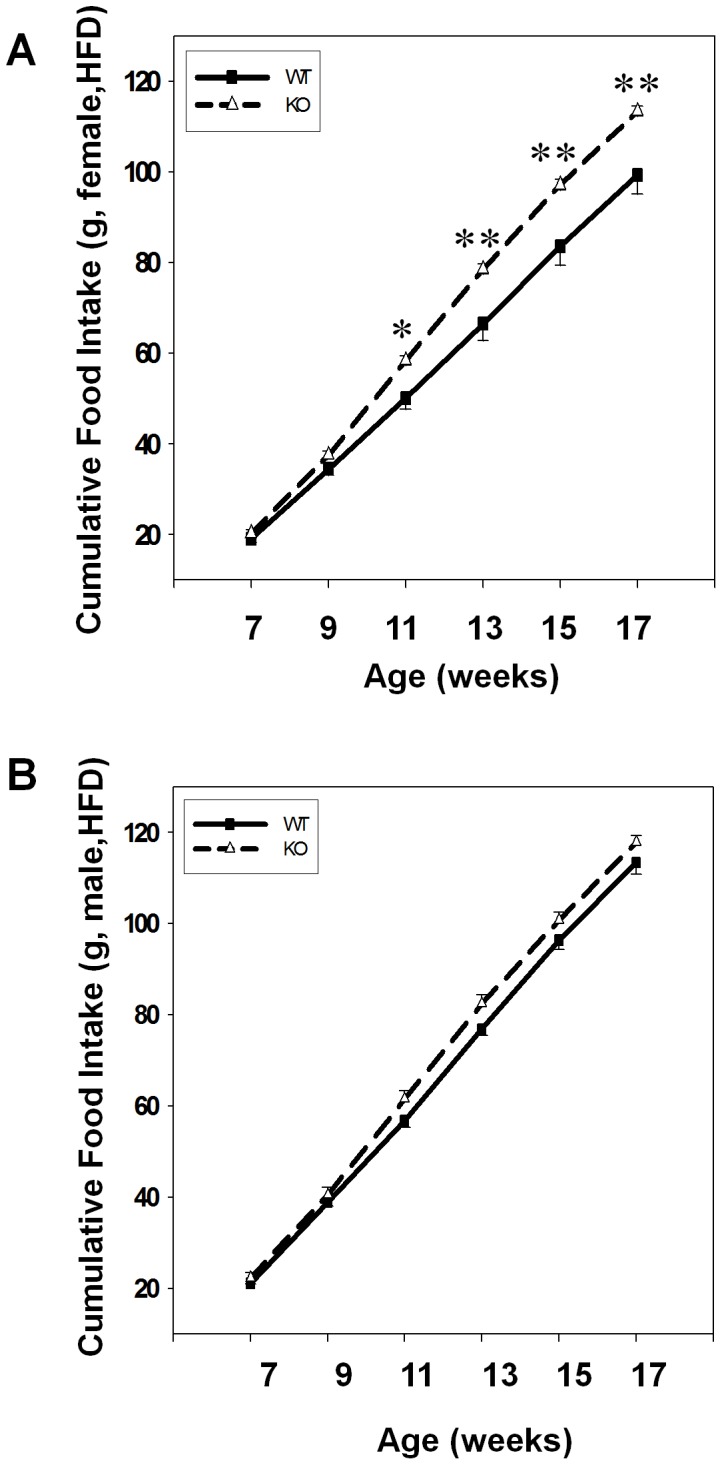
GPR26 deficiency causes hyperphagia. Cummulative food intake was monitored during 12-weeks of high-fat diet from female (**A**) and male (**B**) GPR26 knockout mice (KO) and their wild type littermates (WT). N = 8, *p<0.05, **p<0.01 when compared with wild type controls.

To determine the mechanisms by which GPR26 accelerates diet-induced obesity in GPR26^−/−^ mice, we next analyzed changes in food intake during the 12 weeks period of feeding with a high-fat diet (HFD). As shown by Fig. 4A, cumulative food intake was significantly higher in the female GPR26^−/−^ mice during the 12 weeks of high fat feeding when compared with the wild type controls, implicating a role of GPR26 in regulating appetite. In contrast, there were no significant differences in accumulated food intake between the male GPR26^−/−^ mice and the same gender wild type controls (Fig. 4B). The results suggest that the obese phenotype in female GPR26^−/−^ mice is likely caused by hyperphagia.

**Figure 5:**
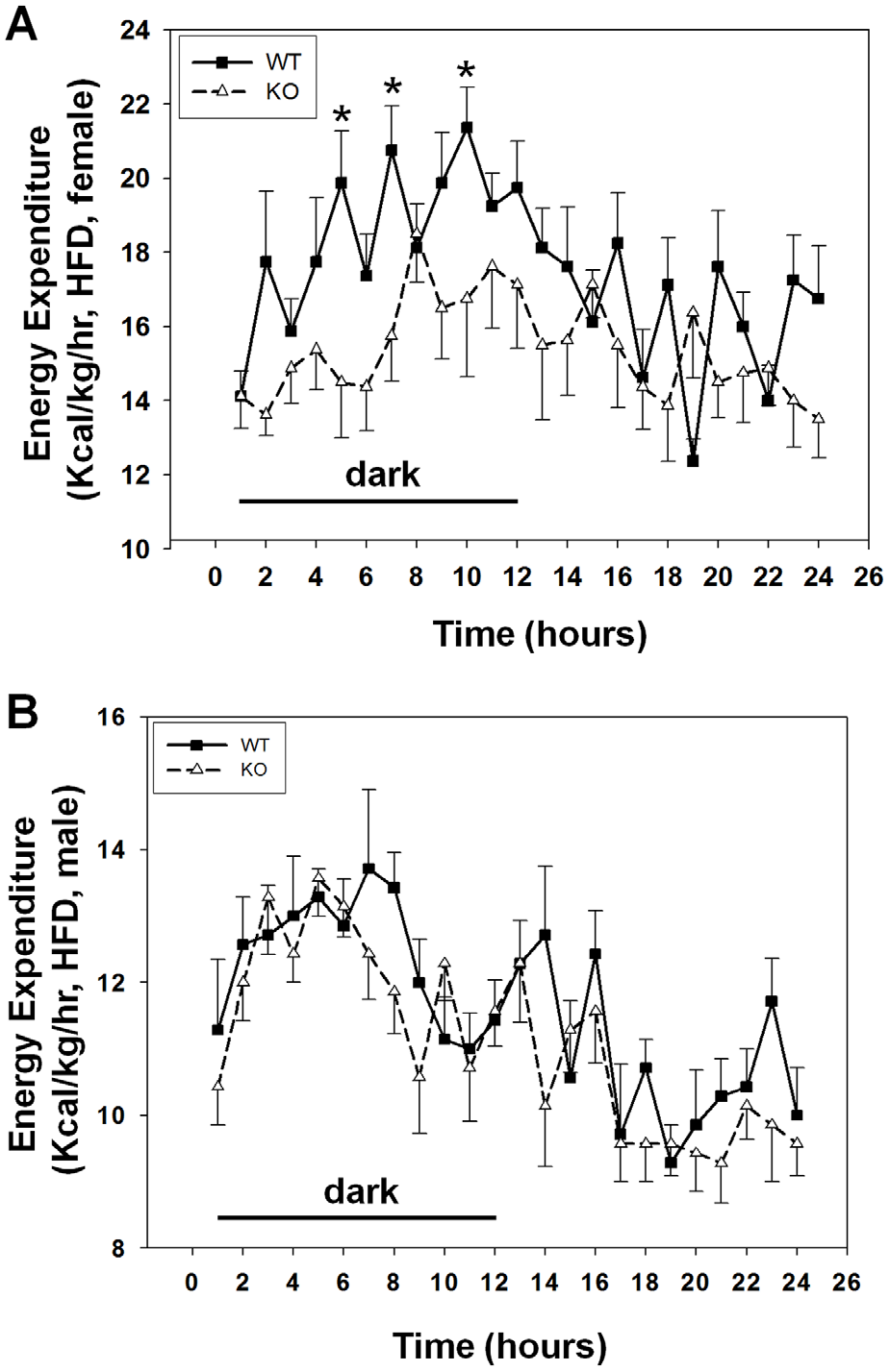
GPR26 deficiency decreased energy expenditure. Energy expenditure were analyzed from GPR26 knockout mice (KO) and wild type littermates (WT) after 12-weeks of high-fat diet using an open circuit calorimetry system (Oxymax). The mice were housed in metabolic cages under a 12-hour light/dark cycle. (**A**): female mice; (**B**): male mice. N = 8 *p<0.05 when compared with wild type controls.

Hyperphagia and increased adiposity in GPR26^−/−^ mice prompted us to investigate a role of GPR26 in regulating energy expenditure by a comprehensive lab animal monitoring system (CLAMS) equipped with an open circuit calorimetry system (Oxymax) [11], [12]. As shown in Fig. 5A, GPR26 deficiency significantly decreased energy expenditure during the dark period without affecting that in the light cycle. In contrast, the wild type control mice exhibit a normal diurnal variation, with higher energy expenditure during the dark period and lower energy expenditure during light cycle, which is consistent with mouse being nocturnal animals. Again, GPR26 deficiency in male mice did not significantly affect energy expenditure (Fig. 5B). The results suggest a role of GPR26 in regulating energy expenditure, which is likely to further exacerbate the obese phenotype in female GPR26^−/−^ mice.

### GPR26 Deficiency Exacerbates Obesity-related Metabolic Complications

**Figure 6:**
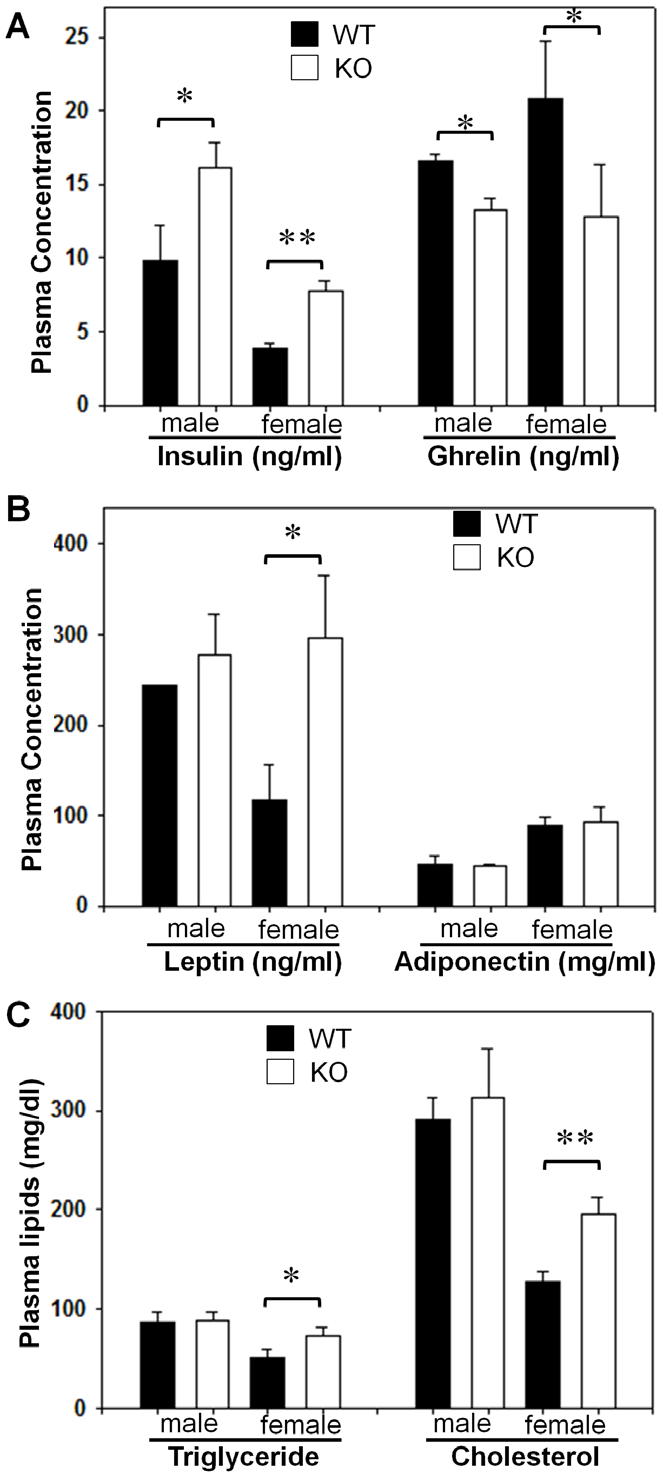
GPR26 deficiency exacerbates hyperinsulinemia and dyslipidemia associated with obesity. After 12-weeks of high-fat diet, GPR26 knockout mice (KO) and wild type littermates (WT) were fasted overnight and blood samples were collected into a tube that contained heparin and EDTA on ice. (**A**–**B**), circulating levels of insulin, ghrelin, leptin, and adiponectin were analyzed by Linco Research service. (**C**), the concentrations of plasma triglyceride and cholesterol were determined using the Roche/Hitachi 912 automatic analyzer system. N = 8, *p<0.05, **p<0.01 when compared with wild type controls.

Obesity is a primary cause of metabolic complications, such as hyperinsulinemia and dyslipidemia. To determine a role of GPR26 in regulating obesity-related metabolic complications, we next analyzed changes in serum levels of insulin, ghrelin, adiponectin, and lipids in GPR26^−/−^ mice on a high-fat diet. As shown in Fig. 6A, GPR26 deficiency significantly increased serum insulin level in both female and male GPR26^−/−^ mice, suggesting hyperinsulinemia in GPR26 knockout mice. Ghrelin is a gastric peptide that stimulates food intake, and circulating ghrelin levels are decreased by the onset of human obesity [12]. Consistent with increased adiposity, serum ghrelin levels were significantly lower in GPR26^−/−^ mice (Fig. 6A). Additionally, GPR26 deficiency also significantly increased serum leptin level in female GPR26 knockout mice (Fig. 6B), which is which is consistent with reported leptinemia in obese patients [13]. In further support a role of GPR26 in metabolic complications, GPR26 also caused dyslipidemia, as evidenced by elevated levels of serum triglyceride and cholesterol in female GPR26^−/−^ mice (Fig. 6C). In contrast, GPR26 deficiency did not significantly affect serum triglyceride level in male mice (Fig. 6C) and adiponectin level in both genders (Fig. 6B), an adipokine which improves insulin sensitivity [14].

### GPR26 Deficiency Stimulates AMPK Activation in the Hypothalamus

**Figure 7:**
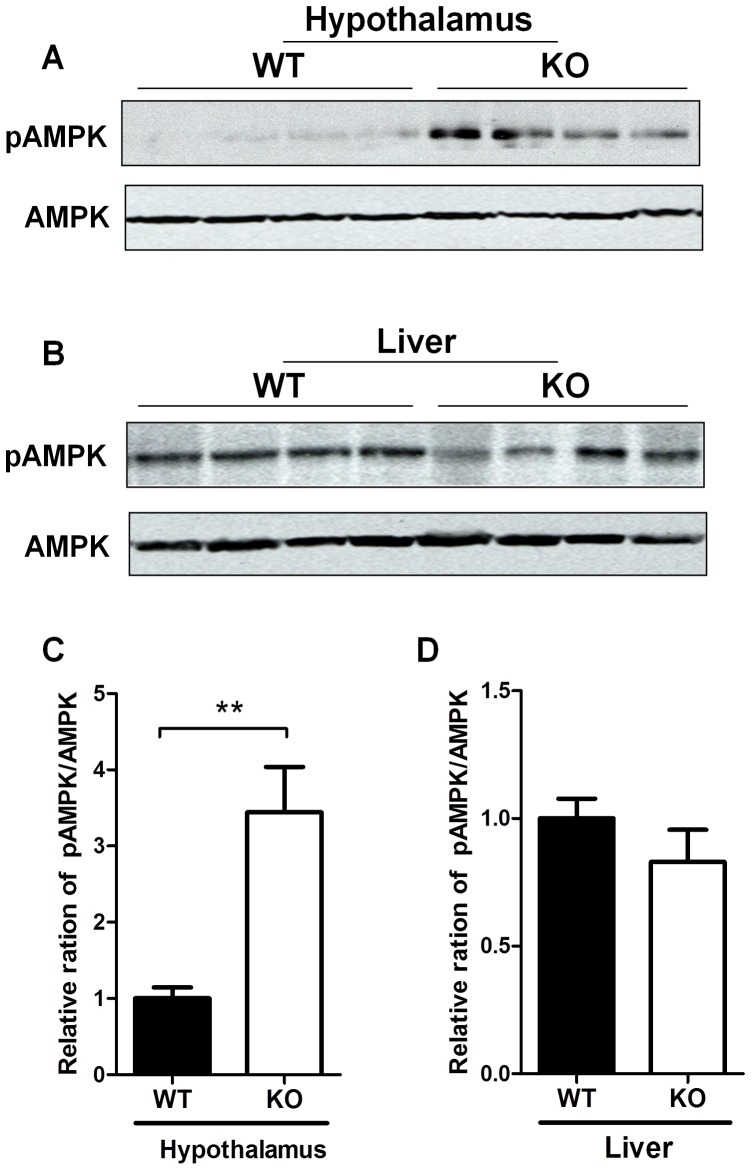
GPR26 deficiency stimulates AMPK phosphorylatoin in the hypothalamus. After 12-weeks of high-fat diet, GPR26 knockout mice (KO) and wild type littermates (WT) were fasted overnight and tissues samples were collected. Western blot analysis was carried out to determine the effect of GPR26 deficiency on phosphorylation of AMPK at Ser172, a key activation site in hypothalamus (**A**) and liver (**B**). (**C–D**), quantitative analysis of AMPK phosphorylation in panel A and B, respectively. N = 4, *p<0.05, **p<0.01 when compared with wild type controls.

To gain further insight into the molecular mechanisms underlying hyperphagia and obesity in GPR26^−/−^ mice, we next analyzed consequence of GPR26 deficiency on AMPK signaling in the brain. AMPK is an energy sensor that is activated in response to glucose deprivation, leading to catabolism in order to restore energy balance [15], [16]. Central AMPK activation has been shown to cause hyperphagia and adiposity [17], [18]. Consistent with hyperphagia in GPR26^−/−^ mice, the results show that GPR26 deficiency significantly stimulated phosphorylation of AMPK at Ser172, a key activation site which is phosphorylated by LKB1 [19], [20], in the hypothalamus of the GPR26^−/−^ mice (Fig. 7A, quantified in Fig. 7C). In contrast, GPR26 deficiency did not significantly affect AMPK phosphorylation at Ser172 in the liver (Fig. 7B, quantified in Fig. 7C). The results are consistent with the previous reports that GPR26 is exclusively expressed in brain [8], [9].

### GPR26^−/−^ Mice Exhibit Hypersensitivity to Weigh Loss Effect of CB1 Antagonist

**Figure 8:**
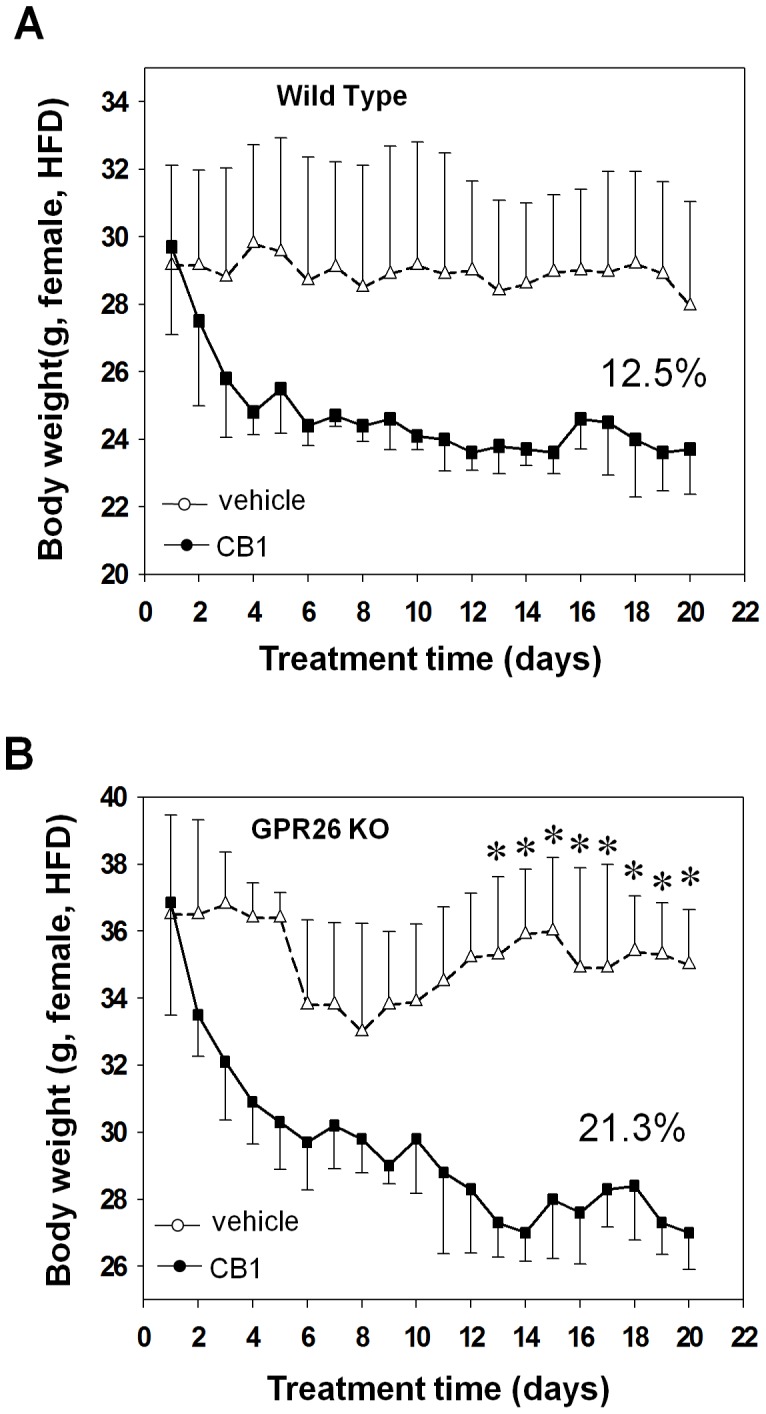
GPR26^−/−^ mice exhibit hypersensitivity to CB1 antagonist. After 12-weeks of high-fat diet, GPR26 knockout mice (KO) and wild type littermates (WT) were administrated daily by oral gavage with 10 mg of rimonabant (SR141716A ), an CB1 antagonist, per kg body weight formulated in 100 µl sodium carboxymethylcellulose (NaCMC) for 20 consecutive days. The same volume of NaCMC was utilized as the vehicle control. (**A–B**), body weight profile of WT (**A**) and KO (**B**) mice during the 20 days of treatment. N = 5, *p<0.05 when compared with wild type controls.

It has previously been shown that GPR26 shares a striking similar tissue distribution with CB1 receptor in central nerve system [8], [15], thus raising an intriguing question whether they are functionally related. The endocannabinoid system in the hypothalamus plays a key role in energy homeostasis by regulating appetite, as exemplified the development of rimonabant, a CB1 receptor antagonist commonly used for the treatment of human obesity. The generation of GPR26 knockout mice affords us the unique opportunity to test this hypothesis. We determined whether GPR26 deficiency would affect the weight loss effect of rimonabant. As shown in Fig. 8A, treatment of wild type control mice for 20 consecutive days resulted in 10% decrease in body weight. In contrast, GPR26 deficiency significantly increased the sensitivity to the weight loss effect of rimonabant, as evidenced by more than 21% weight loss in female GPR26^−/−^ mice in response to the same treatment (Fig. 8B), suggesting a hypersensitivity to rimonabant.

## Discussion

GPR26 is an orphan GPCR whose natural ligand remains to be identified. GPR26 is exclusively expressed in brain, and exhibits the highest sequence homology with the serotonin receptor 5-HT_5A_ and gastrin releasing hormone BB2 receptor, suggesting a potential role in regulating energy metabolism. The GPR26 mRNA is most abundantly expressed in the brain region associated with appetite control [9], and the human GPR26 gene has been mapped to an obesity locus on chromosome 10 q26 [21]. Despite the circumstantial evidence, a potential role of GPR26 in energy metabolism has not been investigated. In the present study, we investigated a role of GPR26 in regulating the onset of diet-induced obesity and its related metabolic complications in mice with targeted deletion of the *GPR26* gene. Our data showed that GPR26-decificent mice exhibit hyperphagia concurrently with decreased energy expenditure, leading to early onset of diet-induced obesity. The defect was more pronounced in female mice. Although the molecular mechanisms underlying gender differences remain to be elucidated in the future, this kind of bias has been reported in other rodent model of obesity [22], [23]. The increased adiposity was further confirmed by QMR scanning which revealed increased fat mass in GPR26 knockout animals. Consistent with increased adiposity, GPR26-decificent mice also developed co-morbidities commonly associated with obesity, including hyperinsulinemia, hyperleptinemia, and dyslipidmia which are known to cause insulin resistance. Accordingly, GPR26 exhibited poor glycemic controls during glucose tolerance test. Our findings are supported by previous reports that depletion of the GPR26 homolog in *C. elegans* significantly increased fat deposition in worms [11], and GPR26 expression level in the hypothalamus is negatively correlated with susceptibility to onset of obesity in mice [24].

Consistent with a key role of GPR26 in regulating energy homeostasis, the present study identified a role of GPR26 in regulating AMPK activation in the hypothalamus. AMPK is an energy sensor, and plays an important role in regulating energy homeostasis [15], [16], [25]. AMPK in the hypothalamus also plays a key role in regulating appetite [18], [26]. Hence, central administration of an AMPK activator AICAR (5-aminoimidazole-4-carboxamide-1-β-D-ribofuranoside) by i.c.v. into the paraventricular nucleus of the hypothalamus significantly increased food intake [17]. Likewise, injection of leptin, an adipokine which potently suppress appetite, into hypothalamus decreased AMPK activity in arcuate nucleus. Furthermore, other anorexigenic signals, including insulin, glucose, and refeeding, also dramatically suppressed AMPK activity in ventromedial/dorsomedial and lateral hypothalamus. Moreover, central administration of constitutively-active AMPK (CA-AMPK) dramatically increased food intake and adiposity [27], whereas injection of Ad-DN AMPK or infusion of an AMPK inhibitor into the hypothalamus led to a significant suppression of glucose production [28]. Accordingly, we show that GPR26 deficiency significantly stimulated phosphorylation of AMPK in the hypothalamus of GPR26 knockout mice. Consistent with exclusive expression of GPR26 in the brain, GPR26 did not affect AMPK phosphorylation in the liver which plays an important role in suppressing hepatic gluconeogenesis and insulin resistance [29]–[31].

Although the molecular mechanism underlying a regulatory role of GPR26 in hypothalamic AMPK activity remains to be elucidated in future studies, it can be envisaged that GPR26 could regulate AMPK activation in both direct and indirect pathways. In the direct pathway, GPR26 activation is known to stimulate the production of intracellular cAMP which has been shown to regulate multiple phosphorylation sites of α subunit of AMPK, including α-Thr^172^ and α1-Ser^485^/α2-Ser^491^, in the clonal INS-1 β-cell [32]. Since islet β-cells and neurons share common molecular pathways in stimulus-secretion coupling, a similar mechanism is likely to exist in hypothalamic neurons involved in regulation of satiety. Alternatively, the activation of AMPK may be a result of feedback response to changes in levels of several of the hormones in GPR26 knockout mice, including insulin, leptin, adiponectin, and ghrelin, all of which play a key role in regulating neuronal AMPK activation in the hypothalamus [33].

In further support of a key role of GPR26 in regulating appetite, the GPR26 knockout mice exhibit hypersensitivity to treatment with rimonabant, an endocannabinoid receptor-1 antagonist commonly used to treat obesity by suppressing appetite in humans [34]. In large randomized clinical trials, rimonabant has been shown to significantly reduce body weight concurrently with an improved metabolic profile of atherogenic dyslipidemia in high-risk patients who are overweight or obese [34]. Consistent with our findings, GPR26 exhibits striking similarity in mRNA expression pattern with that of CB1 receptor [9], [35]. However, rimonabant was recently withdrawn from the market due to unexpected side effect on increased suicidal rate [36], [37]. Therefore, the results from the present studies suggest that targeting GPR26 with chemical activators may provide a novel treatment for obesity through modulation of appetite without the potential side effect of rimonabant. This hypothesis is supported by a recent report that GPR26 deficiency in mice causes increased anxiety and depression [38]. Consequently, a GPR26 activator could also be used for the treatment of depression and anxiety which are often associated with an increased risk of obesity [39].

## Materials and Methods

### Ethics Statement

All animal experiments in this study were conducted in compliance with approved institutional animal care and use protocols according to National Institutes of Health guide lines (NIH Publication No. 86-23), and an animal protocol (protocol number: #2006-053) was approved by the Institutional Animal Care and Use Committee (IACUC) of Penn State University College of Medicine for this study.

### Animal Care

All experiments were performed in F2 (C57BL/6 background) mice. Mice were maintained on a 12-hour light and 12-hour dark cycle. The animals were individually housed at 7 weeks of age and received standard mouse chow (Purina 5015 chow, Ralston Purina Co., St. Louis, MO) or high fat-chow (TD 95217, 40% calories from fat; Teklad, Madison, WI). Body weight and food consumption were measured once a week. Fat and lean body masses were analyzed by quantitative nuclear magnetic resonance (QMR) using an Echo System instrument (Houston, TX), as previously described [22], [23].

### Generation of GPR26 Knockout Mice

A targeting vector was engineered by inserting a 3.2-kb *EcoR* V fragment that contains the 5′ promoter region and a 9.2-kb *EcoR* I fragment that contains genomic sequences downstream from the first exon of GPR26 (Fig. 1B). The targeting vector was linearized and introduced into ES-C57BL/6 mouse embryonic stem cells (ATCC) by electroporation, followed by selection for resistance to G418 and gancyclovir. Clones carrying targeted disruption of the *GPR26* gene were identified by Southern blot analysis using a 5′ external probe on *EcoR* V digested DNA, which yielded the predicted 8.8 kb band for the wild type and 4.4 kb band for the mutant allele. The positive founder mice were initially screened by PCR amplification using primer pair 36662f (tcacagttcttcagatctctaggc) and neo6R (catagccgaatagcctctcc) for targeting at the 5′ end. A PCR product of ∼3.8 kb was generated in heterozygous and knockout mice. The primer pair neo1746F (cctgccatagcctcaggttactc) and 52595r (ctgacccagagctcagtggagcagc) was used to check for targeting at the 3′ end. A PCR product of ∼9.2 kb was generated in heterozygous and knockout mice. The positive founder mice were further confirmed by Southern blot using a 3′ external probe on *EcoR* I digested DNA, which yielded a predicated 10.5 kb band for wild type and 12.5 kb band for the mutant allele.

### Treatment with Rimonabant

Mice on high-fat diet with same gender, age, and genetic background (wild type or GPR26 knockout) were randomized into treatment and control groups respectively. The animals were daily administrated by oral gavage with 10 mg of rimonabant, also known as SR141716A, a CB1 antagonist, per kg body weight formulated in 100 µl sodium carboxymethylcellulose (NaCMC). The same volume of NaCMC was utilized as the vehicle control. Body weight and food intake were monitored daily for 20 consecutive days.

### Oral Glucose Tolerance Test (OGTT)

OGTT was initiated by orally administrating 2.0 g of glucose per kg of body weight. Mouse tail blood samples were collected at the time points of 0, 30, 60, 120 minutes and analyzed for blood glucose levels by using a ACCU-CHEK Blood Glucose Meter (Roche Diagnostics, Indianapolis, IN).

### Body Composition, Energy Expenditure, Activity, and Food Intake

Body fat and lean body mass were measured using LF90 TD-NMR (Bruker Optics). Measurements of food intake, energy expenditure, and respiratory exchange ratio were performed using metabolic cages equipped with a comprehensive lab animal monitoring system (CLAMS) (TSE Systems, Bad Homburg, Germany). Constant airflow (0.4 l/min) was drawn through the chamber and monitored by a mass-sensitive flow meter. The concentrations of oxygen and carbon dioxide were monitored at the inlet and outlet of the sealed chambers to calculate oxygen consumption and respiratory quotient (RQ). Each chamber was measured for 1 min at 15 min intervals.

### Analysis of Circulating Serum Metabolic Biomarkers

Mice were fasted overnight and heart blood was drawn into both heparinized and EDTA tubes on ice. Following centrifugation at 2000 rpm for 5 minutes to obtain plasma, the concentrations of blood triglyceride and cholesterol were determined using the Roche/Hitach 912 automatic analyzer system. Insulin, leptin, ghrelin and adiponectin were measured by Linco Research (St. Charles, MO).

### Western Blotting Analysis

The mouse hypothalamus and liver tissues were rinsed twice in PBS and homogenized in a lysis buffer containing 1% Triton X-100 and protease inhibitors. The protein content was determined using a bicinchoninic acid protein assay kit (Pierce, Rockford IL). Proteins (20 µg) were separated by SDS-PAGE on 10% polyacrylamide gels and then transferred to nitrocellulose membrane. Following blocking of nonspecific binding by 5% nonfat milk, the membranes were probed with a phosphor (p)-AMPK (Thr 172) antibody (Cell Signaling, Danvers MA) at 4°C for 12 hours. The membranes were then washed and incubated with HRP-conjugated, goat anti-Rabbit IgG (1∶5000; DAKO, Carpinteria, CA) and developed using western blot chemiluminescence reagent (NEN Life Science Products, Boston, MA). Following demonstration of p-AMPK, the nitrocellulose membranes were stripped by treatment with a stripping buffer (Pierce) for 30 min and total AMPK level was detected in the same membranes using a rabbit anti-AMPK antibody (Cell Signaling). Densitometry was performed for standardization of the phosphorylated AMPK isoform relative to the total AMPK.

### Statistical Analysis

Statistical comparisons were done using two-tailed non-paired *t*-test to determine difference between GPR26^−/−^ and wild type mice. Analysis of covariance (ANCOVA) was also used to assess differences between GPR26^−/−^ and wild type mice in metabolic parameters relative to total body weight and lean mass. Values are presented as means ± SEM; p<0.05 was considered significantly different.

## References

[1] Rejeski WJ, Ip EH, Bertoni AG, Bray GA, Evans G (2012). Lifestyle change and mobility in obese adults with type 2 diabetes.. N Engl J Med.

[2] Stein CJ, Colditz GA (2004). The epidemic of obesity.. J Clin Endocrinol Metab.

[3] Kahn BB, Flier JS (2000). Obesity and insulin resistance.. J Clin Invest.

[4] Farrigan C, Pang K (2002). Obesity market overview.. Nat Rev Drug Discov.

[5] Jandacek RJ, Woods SC (2004). Pharmaceutical approaches to the treatment of obesity.. Drug Discov Today.

[6] Overton HA, Babbs AJ, Doel SM, Fyfe MC, Gardner LS (2006). Deorphanization of a G protein-coupled receptor for oleoylethanolamide and its use in the discovery of small-molecule hypophagic agents.. Cell Metab.

[7] Vassilatis DK, Hohmann JG, Zeng H, Li F, Ranchalis JE (2003). The G protein-coupled receptor repertoires of human and mouse.. Proc Natl Acad Sci U S A.

[8] Xu YL, Jackson VR, Civelli O (2004). Orphan G protein-coupled receptors and obesity.. Eur J Pharmacol.

[9] Lee DK, Lynch KR, Nguyen T, Im DS, Cheng R (2000). Cloning and characterization of additional members of the G protein-coupled receptor family.. Biochim Biophys Acta.

[10] Bresnick JN, Skynner HA, Chapman KL, Jack AD, Zamiara E (2003). Identification of signal transduction pathways used by orphan g protein-coupled receptors.. Assay Drug Dev Technol.

[11] Ashrafi K, Chang FY, Watts JL, Fraser AG, Kamath RS (2003). Genome-wide RNAi analysis of Caenorhabditis elegans fat regulatory genes.. Nature.

[12] Tschop M, Weyer C, Tataranni PA, Devanarayan V, Ravussin E (2001). Circulating ghrelin levels are decreased in human obesity.. Diabetes.

[13] Myers MG, Leibel RL, Seeley RJ, Schwartz MW (2010). Obesity and leptin resistance: distinguishing cause from effect.. Trends Endocrinol Metab.

[14] Kadowaki T, Yamauchi T, Kubota N, Hara K, Ueki K (2006). Adiponectin and adiponectin receptors in insulin resistance, diabetes, and the metabolic syndrome.. J Clin Invest.

[15] Hardie DG (2007). AMPK and SNF1: Snuffing Out Stress.. Cell Metab.

[16] Zhang BB, Zhou G, Li C (2009). AMPK: an emerging drug target for diabetes and the metabolic syndrome.. Cell Metab.

[17] Andersson U, Filipsson K, Abbott CR, Woods A, Smith K (2004). AMP-activated protein kinase plays a role in the control of food intake.. J Biol Chem.

[18] Kola B (2008). Role of AMP-activated protein kinase in the control of appetite.. J Neuroendocrinol.

[19] Lizcano JM, Goransson O, Toth R, Deak M, Morrice NA (2004). LKB1 is a master kinase that activates 13 kinases of the AMPK subfamily, including MARK/PAR-1.. EMBO Journal.

[20] Zhou G, Myers R, Li Y, Chen Y, Shen X (2001). Role of AMP-activated protein kinase in mechanism of metformin action.. J Clin Invest.

[21] Dong C, Wang S, Li WD, Li D, Zhao H (2003). Interacting genetic loci on chromosomes 20 and 10 influence extreme human obesity.. Am J Hum Genet.

[22] Chen Y, Hu C, Hsu CK, Zhang Q, Bi C (2002). Targeted disruption of the melanin-concentrating hormone receptor-1 results in hyperphagia and resistance to diet-induced obesity.. Endocrinology.

[23] Li J, Romestaing C, Han X, Li Y, Hao X (2010). Cardiolipin remodeling by ALCAT1 links oxidative stress and mitochondrial dysfunction to obesity.. Cell Metab.

[24] Koza RA, Nikonova L, Hogan J, Rim JS, Mendoza T (2006). Changes in gene expression foreshadow diet-induced obesity in genetically identical mice.. PLoS Genet.

[25] Kahn BB, Alquier T, Carling D, Hardie DG (2005). AMP-activated protein kinase: ancient energy gauge provides clues to modern understanding of metabolism.. Cell Metab.

[26] Xue B, Kahn BB (2006). AMPK integrates nutrient and hormonal signals to regulate food intake and energy balance through effects in the hypothalamus and peripheral tissues.. J Physiol.

[27] Minokoshi Y, Alquier T, Furukawa N, Kim YB, Lee A (2004). AMP-kinase regulates food intake by responding to hormonal and nutrient signals in the hypothalamus.. Nature.

[28] Yang CS, Lam CK, Chari M, Cheung GW, Kokorovic A (2010). Hypothalamic AMP-activated protein kinase regulates glucose production.. Diabetes.

[29] Lee JM, Seo WY, Song KH, Chanda D, Kim YD (2010). AMPK-dependent repression of hepatic gluconeogenesis via disruption of CREB.CRTC2 complex by orphan nuclear receptor small heterodimer partner.. J Biol Chem.

[30] Shaw RJ, Lamia KA, Vasquez D, Koo SH, Bardeesy N (2005). The kinase LKB1 mediates glucose homeostasis in liver and therapeutic effects of metformin.. Science.

[31] Li Y, Xu S, Mihaylova MM, Zheng B, Hou X (2011). AMPK phosphorylates and inhibits SREBP activity to attenuate hepatic steatosis and atherosclerosis in diet-induced insulin-resistant mice.. Cell Metab.

[32] Hurley RL, Barre LK, Wood SD, Anderson KA, Kemp BE (2006). Regulation of AMP-activated protein kinase by multisite phosphorylation in response to agents that elevate cellular cAMP.. J Biol Chem.

[33] Sandoval DA, Obici S, Seeley RJ (2009). Targeting the CNS to treat type 2 diabetes.. Nat Rev Drug Discov.

[34] Despres JP, Golay A, Sjostrom L (2005). Effects of rimonabant on metabolic risk factors in overweight patients with dyslipidemia.. N Engl J Med.

[35] Kola B, Hubina E, Tucci SA, Kirkham TC, Garcia EA (2005). Cannabinoids and ghrelin have both central and peripheral metabolic and cardiac effects via AMP-activated protein kinase.. J Biol Chem.

[36] Di Marzo V, Despres JP (2009). CB1 antagonists for obesity–what lessons have we learned from rimonabant?. Nat Rev Endocrinol.

[37] King A (2010). Prevention: Neuropsychiatric adverse effects signal the end of the line for rimonabant.. Nat Rev Cardiol.

[38] Zhang LL, Wang JJ, Liu Y, Lu XB, Kuang Y (2011). GPR26-deficient mice display increased anxiety- and depression-like behaviors accompanied by reduced phosphorylated cyclic AMP responsive element-binding protein level in central amygdala.. Neuroscience.

[39] Atlantis E, Goldney RD, Wittert GA (2009). Obesity and depression or anxiety.. BMJ.

