# Correction: Targeted Inactivation of GPR26 Leads to Hyperphagia and Adiposity by Activating AMPK in the Hypothalamus

**DOI:** 10.1371/annotation/3629b4c2-4810-4bbd-ae13-53d3d01bce60

**Published:** 2012-08-09

**Authors:** Daohong Chen, Xiaolei Liu, Weiping Zhang, Yuguang Shi

There is an error in the order of affiliations for author Xiaolei Liu. Xiaolei Liu's primary affiliation is 2. Department of Pharmacology, School of Pharmaceutical Sciences Central South University, Changsha, China, and their secondary affiliation is 1. Department of Cellular and Molecular Physiology, Pennsylvania State University College of Medicine, Hershey, Pennsylvania, United States of America. Equal contribution is correct.

